# Cardiovascular imaging 2016 in the International Journal of Cardiovascular Imaging

**DOI:** 10.1007/s10554-017-1111-5

**Published:** 2017-03-18

**Authors:** Johan H. C. Reiber, Johan De Sutter, Paul Schoenhagen, Arthur E. Stillman, Nico R. L. Vande Veire

**Affiliations:** 1 Department of Radiology, Division of Image Processing, Leiden University Medical Center, Leiden, The Netherlands; 2 Department of Cardiology, AZ Maria Middelares Gent and University Gent, Ghent, Belgium; 3 Department of Radiology, The Cleveland Clinic Foundation, Cleveland, OH USA; 4 Department of Radiology, Emory University Hospital, Atlanta, GA USA; 5 Department of Cardiology, AZ Maria Middelares Gent and Free University Brussels, Brussels, Belgium

## Introduction

An overview of the most relevant papers in the International Journal of Cardiovascular Imaging over the year 2016 for the different modalities. Relatively few manuscripts in the field of X-ray imaging were published in 2016. The far majorities were in MSCT, MRI and echocardiography, followed by nuclear cardiology and intravascular imaging, of which the last one is not included in this overview.

## X-ray imaging

Author He et al. described the use of the Myocardial Perfusion Frame Count (TMPFC) technique as an indicator to predict left ventricular systolic dysfunction in the sub-acute phase of STEMI [1]. They counted the number of frames from the first appearance of myocardial blush beyond the infarct related artery until the frame when contrast or myocardial blush disappeared; frame rate was either 15 or 30 f/sec. The ROC curves identified a cut-off of 94 frames for TMPFC to differentiate between normal and abnormal wall motion score index by echo-strain parameters in the subacute phase of STEMI. A randomized comparison of fluoroscopic techniques for implanting pacemaker leads on the right ventricular outflow tract (RVOT) septum was described by Chen et al. [2]. |They evaluated the accuracy of the combination of standard fluoroscopic and left lateral (LL) fluoroscopic views for the determination of RVOT septal positions compared with standard views alone in a total of 143 patients. They found that the success rate of RVOT septal position in the group with three standard fluoroscopic views plus left lateral fluoroscopic views was significantly higher than in the standard group (p = 0.029).

The team of Starek et al. tested various acquisition protocols for the right atrium and the left atrium to create 3D models of the atria and esophagus using 3D rotational angiography [3]. They concluded that 3D rotational angiography is a reliable method that supports catheter ablation of complex atrial arrhythmias. The left atrial protocol with esophagus imaging was significantly more reliable than the right atrial protocol, which may be negatively affected by high BMI. Another rotational angiography based paper was published by Rodriguez-Olivares et al., in which they studied the determinants of image quality for on-line assessment of frame geometry after TAVI [4]. They used dedicated research prototype software for motion compensation without rapid ventricular pacing after the implantation of four commercially available catheter-based valves. They concluded that this specific solution with motion compensation offers good image quality, bit it was negatively affected by certain valve types, presence of artefacts and higher BMI.

Another topic that received attention was longitudinal stent deformation and the longer term clinical outcomes [5]. Guler et al. concluded that the use of extra-support guiding catheter, extra-support guidewires and low stent inflation pressure increases the occurrence of longitudinal stent deformation. But with proper treatment, unwanted long-term outcomes can be successfully prevented.

Timmins et al. compared in a very small cohort of patients (n = 5) with non-obstructive coronary artery disease biplane angiographic versus intravascular ultrasound derived reconstructed coronary geometries, assessed wall shear stress and the association of wall shear stress and CAD progression after 6 months [6]. They concluded from this limited set a strong agreement between angiographic and IVUS-derived coronary geometries, but limited agreement was observed between computed wall shear stress values and associations of wall shear stress with CAD progression.

Donmet et al. studied in a population of 81 patients the changes over time in non-culprit lesions in patients wih ST segment elevated myocardial infarction using QCA [7]. They found that in approximately 50% of the cases, the non-culprit lesions were found less critical during control angiography when compared to primary PCI. And therefore, they suggest that complete revascularization during primary PCI should be avoided in multi-vessel STEMI patients and critical non culprit artery lesions should be re-evaluated with later control angiography.

## Nuclear cardiology

In 2016 different excellent paper in the field of nuclear cardiology were published in the journal. In this review we selected a few papers on technical advances, atherosclerosis imaging, imaging in heart failure and FDG PET/CT imaging in infective endocarditis.

### Technical advances

Perfusion SPECT imaging may be technically challenging in obese patients and data on its prognostic value in the obese are rather scarce. De Lorenzo et al. [8] studied 365 obese patients among 1396 patients referred for single-day rest/stress perfusion imaging using a dedicated multipinhole cadium-zinc telluride (CTZ) SPECT camera. Image quality was good/excellent in 94.5% of these obese patients. The annualized mortality rates were not significantly different among obese and non-obese patients, being <1% with normal CTZ-SPECT and increased with the degree of scan abnormality in both obese and non-obese patients. Obesity itself was nog an independent predictor of death. The authors conclude that single-day stress/rest CTZ-SPECT with a multipinhole camera provides prognostic information with high image quality in obese patients.

High efficiency CZT cameras provide also an opportunity to lower the injected activities of radiopharmaceuticals for SPECT myocardial imaging. Kincl et al. [9] evaluated the feasibility of ultra-low dose Thallium stress-redistribution including prone imaging in obese patients using a CZT camera. They studied 124 patients with an ultra-low dose Thallium of 0.5 MBq/kg. The mean administered activity was 39.2 ± 7 MBq for non-obese and 48.7 ± 6 for obese patients (p < 0.0001) and the calculated effective dose was 4.0 ± 0.7 and 4.9 ± 0.6 mSv respectively (p < 0.0001) or a 50% radiation reduction in comparison with previous studies using CZT cameras. Further analysis showed that these ultra-low dose Thallium stress-redistribution protocol, including post-stress prone imaging, provided good quality of images with excellent interobserver variability both in obese and non-obese patients.

### Atherosclerosis imaging

Several excellent reviews on the topics of atherosclerosis and multi-modality imaging were published in 2016. FDG-PET has shown promise in detecting metabolically active inflammatory cell infiltrates associated with vulnerable atherosclerotic plaque. In contrast to previous ex vivo studies incuatong plaque specimens with FDG after excision, Liu et al. [10] investigated which plaque components contribute to enhanced FDG uptake in vivo. They utilized high-resolution micro-PET on carotid plaques excised from 14 patients, injected with FDG just prior to carotid endarterectomy. The results of this very nice study indicate that in vivo uptake of FDG corresponded to regions of inflammatory cell infiltrate in human carotid atherosclerotic plaque, particularly, complex inflammatory cell infiltrated with co-localized macrophages, lymphocytes and foam cells. The use of high-resolution micro-PET also revealed FDG uptake in other plaque components such as loose extracellular matrix and neovasculature. The overlay of FDG-PET signal with serially sectioned and matched histology validated that FDG uptake had good correlation with the active components of the human atherosclerotic plaque.

### Imaging in heart failure

Left ventricular dyssynchrony (LVD) is an independent predictor of adverse cardiovascular events and progression to heart failure. It can also be potentially corrected with cardiac resynchronization therapy. LVD can be diagnosed using phase analysis on myocardial perfusion imaging with ECG-gated SPECT. Tavares et al. [11] evaluated clinical, electrocardiographic and scintigraphic data from 1000 patients who underwent MPI with ECG-gated SPECT. The Emory Cardiac Toolbox software was used for phase analysis and LVD was diagnosed based on the following criteria: standard deviation of LV phase distribution ≥ 43° and/or phase histogram ≥ 140° in the resting and/or stress phase of the examination. Although the phase analysis parameters were greater at rest, both phases could be used for diagnosis. Multivariate analysis revelead that that male sex, obesity, history of CAD and QRS interval ≥ 120 ms were independently associated with LVD. The overall prevalence of LVD was 6.5%, and it reached 42% in the presence of certain risk factors. Although this study clearly indicate that LVD evaluation with nuclear imaging is feasible and frequently present, its prognostic value and impact on device therapy needs further evaluation.

### FDG PET/CT imaging in prosthetic valve endocarditis

Recent studies have shown promising results using (18)F-FDG PET/CT for the diagnosis of prosthetic valve endocarditis (PVE) and the use of this nuclear imaging technique has been recently advocated in American and European guidelines on the management of endocarditis. However, negative controls were usually lacking in these studies. Fagman et al. [12] compared (18)F-FDG uptake around prosthetic aortic valves in 8 patients with definite PVE to the (18)F-FDG uptake in 19 patients with an aortic prosthesis without PVE. Visual analysis showed a sensitivity of 75%, specificity of 87%, positive likelihood ratio of 4.8 and negative likelihood ratio of 0.3. Semi quantitative analysis using maximal standardized uptake values (SUV) in the valve area and in the descending aorta showed an area under the curve of 0.90 (95% CI 0.74–1.0) using ROC-curve analysis for the SUV-ratio. The authors concluded that (18)F-FDG uptake in the prosthetic valve area had an overall good diagnostic performance in the diagnosis of PVE.

## Echocardiography

In the 2016 edition of the International Journal of Cardiovascular Imaging several interesting studies were published in the field of cardiac ultrasound. Some of them are discussed in this overview.

### Grading of diastolic function by echocardiography

The American and European scientific echo community have published an algorithm for the grading of diastolic function. However, the ability to use this algorithm effectively in daily clinical practice has not been investigated. Van Dalen et al. hypothesized that in some patients it may be difficult to grade diastolic dysfunction with this scheme, since there may be discrepancies in the assessed parameters [13]. The ASE/EAE algorithm starts with assessment of diastolic myocardial wall velocities and left atrial volumes with subsequent assessment of E/A ratio, E-wave deceleration time and pulmonary venous flow. The aim of the current study was to test the feasibility of the ASE/EAE algorithm and to compare this with a new “Thoraxcenter” algorithm. The Thoraxcenter algorithm reverses these steps, uses left atrial dimension instead of volume and does not include a Valsalva manoeuvre and pulmonary venous flow. Due to inconsistencies between diastolic myocardial wall velocities and left atrial volumes and a not covered E/A ratio in the range of 1.5–2 it was not possible to classify 48% of patients with the ASE/EAE algorithm, as opposed to only 10% by the Thoraxcenter algorithm. Left atrial volume was always needed in the ASE/EAE algorithm. In only 64% of patients left atrial size was necessary by the Thoraxcenter algorithm. When left atrial volume would have been used instead of left atrial dimension, grading of LV diastolic function would have been different in only 2% of patients without apparent improvement. Assessment of left atrial dimension was considerably faster than left atrial volume. The Thoraxcenter algorithm to grade LV diastolic dysfunction was - compared to the ASE/EAE algorithm - simpler, faster, better reproducible and yields a higher diagnostic outcome.

### Left atrial minimum volume versus left atrial maximum volume to assess left ventricular filling

Previous data have demonstrated that left atrial (LA) minimum volume indexed for body surface area (LAVImin) is more strongly associated with the Doppler echocardiographic E/e′ ratio than LA maximum volume index (LAVImax). Hedberg et al. sought to explore if LAVImin was more closely related to serum levels of NT-proBNP than LAVImax and E/e′ in the community [14]. A community-based sample of 730 subjects underwent echocardiographic examinations and NT-proBNP measurements. Age, LAVImin, LAVImax, estimated glomerular filtration rate and E/e′ were strongly correlated with log-NT-proBNP. In a multiple linear regression model with log-NT-proBNP as dependent variable and LAVImin, LAVImax, E/e′ ratio, and potential confounders as predictors, an adjusted R^2^ of 44.9% was obtained. When excluding either of LAVImin or E/e′ the model fit was significantly reduced. In contrast, when LAVImax was excluded the model fit was preserved. To detect an NT-proBNP level of >125 ng/L, LAVImin yielded a significantly larger area under the receiver operating characteristic curve than LAVImax and E/e′. In this community-based sample, LAVImin was more strongly associated with NT-proBNP than LAVImax. Moreover, the discriminatory power to detect an elevated NT-proBNP level was stronger in LAVImin than in LAVImax and E/e′. These findings support previous data that LAVImin may be more closely related to left ventricular filling function than LAVImax.

### Is 3-dimensional transoesophageal echocardiography superior for the evaluation of mitral valve prolapse ?

De Groot-de Laat et al. assessed the incremental value of two-dimensional and three-dimensional transoesophageal echocardiography over two-dimensional transthoracic echocardiography in three reader groups with different expertise (novice, trainees, cardiologists) in a total of twenty patients and five healthy persons [15]. Overall there was an improvement in agreement and Kappa values from novice to trainees to cardiologists. Diagnostic accuracies of 2D-transoesophageal echocardiography were higher than those of 2D-transthoracic echocardiography mainly in novice readers. Time to diagnosis was dramatically reduced from 2D- to 3D-transoesophageal echocardiography in all reader groups. 3D-transoesophageal echocardiography also improved the agreement (+12 to +16%) and Kappa values (+0.14 to +0.21) in all reader groups for the exact description of P2 prolapse. Differences between readers with variable experience in determining the precise localization and extent of the prolapsing posterior mitral valve scallops exist in particular in 2D- transthoracic echocardiography analysis. 3D-transoesophageal echocardiography analysis was extremely fast compared to the 2D analysis methods and showed the best diagnostic accuracy (mainly driven by specificity) with identification of P1 and P3 prolapse still improving from novice to trainees to cardiologists and provided optimal description of P2 prolapse extent.

### Best approach to evaluate paravalvular regurgitation after transcatheter aortic valve implantation using transthoracic echocardiography

Paravalvular leak after transcatheter aortic valve implantation (TAVI) is challenging to quantitate. Transthoracic echocardiography is the main tool used for the assessment of paravalvular leak but is modestly reproducible. Abdelghani et al. sought to develop a reproducible echocardiographic approach to assess paravalvular leak in the post-TAVI setting [16]. Four observers independently analyzed eleven parameters of paravalvular severity in 50 pre-discharge echo studies performed after TAVI. Inter and intra-observer intraclass correlation coefficients were highest and coefficient of variation lowest for jet circumferential extent, jet origin breadth, jet qualitative features in long-axis views, jet time velocity integral and pressure half time. Combining color Doppler and continuous wave Doppler parameters in a granular algorithm yielded excellent reproducibility of paravalvular leakage assessment by transthoracic echocardiography.

### Global longitudinal strain for the early detection of ventricular dysfunction in patients with repaired aortic coarctation

Despite successful aortic coarctation (CoA) repair, systemic hypertension often recurs which may influence left ventricular function. Menting et al. aimed to detect early left ventricular dysfunction using left ventricular global longitudinal strain (GLS) in adults with repaired CoA, and to identify associations with patient and echocardiographic characteristics [17]. In this cross-sectional study, patients with repaired CoA and healthy controls were recruited prospectively. Left ventricular GLS was lower in patients than in controls (−17.1 ± 2.3 vs. −20.2 ± 1.6%, P < 0.001). Eighty percent of the patients had a normal left ventricular ejection fraction, but GLS was still lower than in controls (P < 0.001). In patients, GLS correlated with systolic and diastolic blood pressure, QRS duration, left atrial dimension, left ventricular mass and left ventricular ejection fraction. Patients with either associated cardiac lesions, multiple cardiac interventions or aortic valve replacement had lower GLS than patients without. Although the majority of adults with repaired CoA seem to have a normal systolic left ventricular function, left ventricular GLS was decreased. Higher blood pressure, associated cardiac lesions, and larger left atrial dimension are related with lower GLS. Therefore, left ventricular GLS may be used as objective criterion for early detection of ventricular dysfunction.

### Longitudinal strain-volume/area relationships in athletes

Oxborough et al. simultaneously assessed longitudinal strain and left ventricular volume/ right ventricular area in 92 male athletes subdivided according to varying sporting demographics [18]. Athletes with a high static–high dynamic profile have greater resting longitudinal contribution to volume change in the left ventricle which, in part, is related to an increased wall thickness. A lower longitudinal contribution to area change in the right ventricle is also apparent in these athletes.

### Contrast-enhanced ultrasound

Schinkel et al. wrote an interesting overview on the role of contrast-enhanced ultrasound (CEUS) in the evaluation of patients with known or suspected atherosclerosis [19]. CEUS is a high-resolution, noninvasive imaging modality, which is safe and may benefit patients with coronary, carotid, or aortic atherosclerosis. The administration of a micro-bubble contrast agent in conjunction with ultrasound results in an improved image quality and provides information that cannot be assessed with standard B-mode ultrasound. CEUS allows a reliable assessment of endocardial borders, left ventricular function, intracardiac thrombus and myocardial perfusion. CEUS results in an improved detection of carotid atherosclerosis, and allows assessment of high-risk plaque characteristics including intra-plaque vascularization, and ulceration. CEUS provides real-time bedside information in patients with a suspected or known abdominal aortic aneurysm or aortic dissection. The absence of ionizing radiation and safety of the contrast agent allow repetitive imaging which is particularly useful in the follow-up of patients after endovascular aneurysm repair. New developments in CEUS-based molecular imaging will improve the understanding of the pathophysiology of atherosclerosis and may in the future allow to image and directly treat cardiovascular diseases (theragnostic CEUS). Familiarity with the strengths and limitations of CEUS may have a major impact on the management of patients with atherosclerosis.

## Magnetic resonance imaging

There were a number of interesting advancements in cardiovascular MRI (CMR) in 2016.

Reval et al. [20] studied patients with left bundle branch block for mechanical features of dyssynchrony and found to more frequently have septal flash, apical rocking, and delayed aortic opening relative to end-diastole and pulmonic valve opening. The delayed aortic valve opening was found to be positively correlated with QRS duration and negatively correlated with ejection fraction. Hu et al. [21] found that the level of Galectin-3 added prognostic value over late gadolinium enhancement (LGE) in patients with non-ischemic cardiomyopathy. Left ventricular wall thickness was compared in patients with hypertrophic cardiomyopathy and echocardiography [22]. Echocardiography was found to measure greater thickness than CMR. Contrast echocardiography was more similar in thickness. Pozo et al. [23] found that early gadolinium enhancement was a common feature in patients with hypertrophic cardiomyopathy even in the absence of late gadolinium enhancement. Wu et al. [24] found that quantitative diffusion-weighted CMR was a feasible alterative to extra cellular volume for characterizing the extent of fibrosis in patients with hypertrophic cardiomyopathy. CMR was found able to detect cardiac involvement in patients with active eosinophilic granulomatosis even when cardiac symptoms were not present [25].

Improved border sharpness of post infarct scar was demonstrated by Rutz et al. [26] with a self-navigator 3D whole heart pulse sequence. In a series of 647 asymptomatic subjects, Nham et al. [27] found that silent myocardial infarction was not independently associated with ventricular mass, geometry and function, whereas it was associated with diabetes mellitus. The feasibility of three dimensional fusion of electromechanical mapping and LGE CMR for real-time intramyocardial cell injections in a porcine model was established [28].

The prognostic value of stenosis class as measured by magnetic resonance angiography over traditional risk factors in patients with peripheral arterial disease was demonstrated by van den Bosch et al. [29]. Improved in vitro visualization of the lumen in peripheral nitinol stents was found using off-resonance magnetic resonance angiography compared with T1-weighted acquisition [30]. Li and Wang [31] found a poor correlation for plaque lipid content to the contralateral carotid artery suggesting local effects for the development of high-risk lesions. Aortic stiffness in patients with systemic lupus erythematosus and rheumatoid arthritis was studied by CMR [32].

Faletti et al. [33] found that CMR could be used reliably for aortic annulus sizing compared with computed tomography and transesophageal echocardiography. The prognostic value of T1-mapping to derive extra-cellular volume was demonstrated in patients receiving transcatheter aortic valve implantation (TAVI) [34]. The blood flow characteristic in the ascending aorta were assess in patients following TAVI and compared with patients following stented aortic bioprostheses [35]. It was found that the latter had significantly more extensive vertical and helical flow patterns than either TAVI or controls.

The performance of an accelerated CMR protocol using iterative SENSE reconstruction and spatio-temporal L_1_-regularization (IS SENSE) was demonstrated [36]. In related work, Bogachkov et al. [37] used this technique for right ventricular assessment. A 3D-Dixon based method was developed for measuring epicardial and pericardial fat volumes [38].

The feasibility of heart deformation analysis for measuring regional myocardial velocity with CMR was tested in normal volunteers [39]. Cardiac strains as measured by applying feature tracking to CMR images were found to be significantly impaired in patients with acute myocarditis [40]. Kawakubo et al. [41] found that a semi-automatic longitudinal strain analysis useful for evaluating LV and RV dysfunction for a variety of pathologies.

Markl et al. [42] performed 4D flow MRI in patients with atrial fibrillation reduced mean velocities and higher stasis compared with controls. In other work, the relationship between left atrial appendage emptying and left atrial function was compared with the presence of left atrial spontaneous echogenic contrast on transesophageal echocardiography in patients with atrial fibrillation [43]. Good qualitative grading of aortic regurgitation was found for 4D flow comparted with echocardiography [44].

A dose correction for post-contrast T1 mapping of the heart was proposed by Gai et al. [45] and applied to subjects in the MESA study. Increased T1 values were found in athletes having ≥ 5 years of sports activity compared with controls suggesting the development of myocardial fibrosis [46]. Increased native T1-values were found at the interventricular insertion points of patients with precapillary pulmonary hypertension [47].

A semi-automated cardiac segmentation tool was found to improve the reproducibility and speed of right heart MRA [48].Knowledge-based reconstruction was found to be useful for CMR volumetry of the right ventricle after arterial switch for dextro-transposition of the great arteries [49]. Ghelani at al [50] compared echocardiography and CMR based strain analysis of functional single ventricles and found good intra-modality reproducibility, but inter-modality was only modest.

## Computed tomography

In 2016 a large number of original articles, covering a variety of topics related to CT, were submitted to the journal and underwent a rigorous review/selection process. While the submission, review, and editorial selection process has well-known biases, the articles published reflect current trends in computed tomography.

### Coronary artery disease

Coronary artery disease is a major focus of cardiac CT. While clinical prevention guidelines suggest a limited role, coronary calcium scoring (CAC) remains a topic of ongoing research. Published papers describe the effective radiation exposure among participants from the MESA cohort [51], and discuss modifications of acquisition technique to allow further dose reduction, e.g. using iterative model based reconstruction [52]. Other studies examined the association between CV risk factors and coronary calcification in different patient populations [53–55].

In contrast to CAC, which identifies only calcified plaque, contrast-enhanced CTA can assess overall plaque burden and plaque characteristics. While it is not an indication for CTA itself, plaque burden is evaluated by experienced clinical readers in coronary CTA indicated for suspected obstructive CAD and is a topic of significant interest in research. Published papers describe a correlation between elevated HBA1c and higher frequency of obstructive CAD and vulnerable atherosclerotic coronary plaque characteristics (positive vessel remodeling and low-attenuation plaques) in patients with type 2 diabetes [56, 57] An outcome study evaluated prognostic value of coronary CTA (composite endpoint of all-cause mortality, nonfatal myocardial infarction, and unstable angina requiring hospitalization) in a cohort of diabetic patients without known CAD, and a control group without diabetes [58]. Multivariate analysis showed significant prognostic value in diabetic patients over Framingham Score for plaque segment involvement score (SIS) and the segment stenosis score (SSS), while Coronary artery calcium score (CACS) did not add prognostic value in this cohort.

Based on the experience with invasive coronary angiography as well as CTA, the limitations of luminal stenosis assessment for the prediction of hemodynamic significance of CAD are well known. CT techniques to evaluate lesion functional significance are therefore a major focus of research. Among them, non-invasive fractional flow reserve measured by coronary computed tomography angiography (FFR_CT_) has gained significant interest. One published study examined the feasibility of FFR_CT_ in a small ‘unselected’ cohort of patients with suspected significant CAD [59]. FFR_CT_could be measured in the majority of consecutive, patients who had suspected significant CAD by CCTA and demonstrated good diagnostic performance for detecting hemodynamically significant CAD even in patients with calcified vessels. Another important study demonstrated the impact of image resolution on geometrical reconstruction and subsequent FFR calculation with invasive and CT FFR [60]. Disagreement was found in 17.5% vessels. The difference between FFRCTA and FFRQCA correlated with the deviation between minimal lumen areas by CCTA and by ICA. Another parameter of functional coronary lesion significance is myocardial perfusion. A published study evaluated the ability of adenosine stress computed tomography perfusion (CTP) findings to predict mid-term major adverse cardiac events (MACE) (cardiac death, non-fatal myocardial infarction and revascularizations) in patients with acute-onset chest pain, but normal electrocardiograms and troponins [61]. After adjustment for the pretest probability of obstructive coronary artery disease, both detection of a PD and stress TPR were significantly associated with MACE. Other studies demonstrated the feasibility of four-dimensional (4D) whole-heart computed tomography perfusion (CTP) of the myocardium [62] and the ability of cardiac CT for the evaluation of myocardial delayed enhancement (MDE) in the assessment of patients with cardiomyopathy, compared to cardiac MRI [63].

Clinical indications for coronary CTA in patients with known advance obstructive disease are limited, but CT has a defined role in the early evaluation of new coronary stent systems. A pre-clinical study evaluated visualization of polymeric bioresorbable scaffolds (BRSs) by micro-computed tomography (mCT) in a coronary bifurcation model [64]. The translucent structure of the bioresorbable scaffold allows evaluation the coronary lumen with coronary CTA. A clinical study evaluated the accuracy of coronary CTA for in-scaffold quantitative evaluation with optical coherence tomography (OCT) as a reference [65]. In the scaffolded segment, coronary CTA underestimated minimal lumen area by about 10%. Another study evaluated prevalence and clinical implication of stent fracture and longitudinal compression in first- and new-generation drug-eluting stents (DES) using coronary computed tomography angiography (CCTA) [66]. Lastly a study compared costs and clinical outcomes of invasive versus non-invasive diagnostic evaluations for patients with suspected in-stent restenosis (ISR) after percutaneous coronary intervention [67].

### Structural and valvular heart disease intervention

An important focus of CT are indications in the context of structural heart disease intervention. For TAVR planning, one study evaluated an automatic aortic root landmarks detection method with automated determination of annulus radius, annulus orientation, and distance from annulus plane to right and left coronary ostia [68]. Other studies describe the use of CT in the context of LAA occlusion. Left atrial appendage (LAA) structure and morphology was compared between real-time three-dimensional transesophageal echocardiography (RT3D-TEE) and enhanced cardiac computed tomography (CT) [69]. Bland–Altman analysis demonstrated that the LAA measurements obtained using RT3D-TEE were lower than those obtained with the CT.

### Software development and data analysis

Exciting progress is described in analysis of CT data. Published studies examined quantitative semi-automated methods for assessment of coronary luminal stenosis severity [70] and fully-automated techniques for the extraction of the entire arterial access route from the femoral artery to the aortic root for TAVR evaluation [71] or the carotid arteries in CTA in the thorax and upper neck region [72]. Other studies examined subtraction CCTA in the evaluation of in-stent restenosis [73] and fusion of 3D echocardiography (3DE) with multidetector computed tomography (MDCT) to correlate territorial longitudinal strain (LS) with coronary stenosis [74]. The role of dual-energy CT angiography (DE-CTA) for imaging of the aorta was evaluated [75]. Other studies described a non-invasive approach for in vivo assessment of endothelial shear stress (ESS) ESS by coronary computed tomography angiography (CTA) potentially allowing combined local hemodynamic and plaque morphologic information for risk stratification in patients with coronary artery disease [76]. Lastly a study investigated the use of artificial neural networks (ANN) to improve risk stratification and prediction of MPI and angiographic results [77].

The above selection of published articles about cardiovascular CT reflects current clinical use and research interests. Use of CT, like any other diagnostic test, has to balance anticipated benefit against potential risk, specifically for CT radiation exposure and contrast administration. This risk assessment takes into account the susceptibility of specific patient populations [78, 79]. The journal will continue to offer a platform for rapid publication of high-quality research in the field of cardiovascular imaging.
